# Analysis of Basketball Technical Movements Based on Human-Computer Interaction with Deep Learning

**DOI:** 10.1155/2022/4247082

**Published:** 2022-04-14

**Authors:** Xu-Hong Meng, Hong-Ying Shi, Wei-Hong Shang

**Affiliations:** Basic Teaching Department, Hebei Vocational University of Industry and Technology, Shijiazhuang 050091, China

## Abstract

With the continuous development of computer technology, analysis techniques based on various types of sports data sets are also evolving. One typical representative is image-based motion recognition technology, which enables video action recognition with a certain degree of feasibility. In basketball technical action videos, technical action has obvious characteristics. The athletes in the footage in sports videos are relatively fixed, and the scenes are relatively homogeneous, so technical action analysis of basketball technical action videos has certain advantages. However, there are many challenges in basketball technical action recognition, mainly including the fact that basketball techniques are numerous and complex. To address the above issues, this research proposes a 3D convolutional neural network framework that two different resolution image inputs are performed on the basketball technical action dataset. The experimental results show that the algorithmic process designed in this study is effective for action recognition on the basketball technical action dataset.

## 1. Introduction

In recent years, with the awakening of the population's awareness of physical fitness, various sports have been promoted and popularized. In China, basketball is one of the most popular sports for young people between the ages of 20 and 35. The equipment is simple, and a single court can accommodate 10–20 people. In addition, basketball is an all-round exercise for the human body. Smaller players can use their speed to move through the crowd, while bigger players can use their physical strengths to play on the back. Thus, no matter what physical condition an athlete is in, he or she can find the right position on the court. At the same time, basketball can be not only a one-on-one game but also a competitive team sport, where the sense of achievement of a beautifully organized attacking score is not dissimilar to that of a great dummy pass.

With the upgrade of mobile devices and the rise of the video industry, more and more athletes are focusing on using video to analyze their skills during basketball training [1, 2]. At the same time, recognition and classification of image data and even video data have become possible with the increase in GPU computing power and the proposal of excellent models in the field of deep learning [3–5]. As an example, models based on convolutional neural networks have an important place in deep learning [6–9]. In addition, there are many other deep learning algorithms that are constantly being developed, such as BP neural network algorithm [10, 11], back-gradient algorithm [12], generative adversarial network [13, 14], transformer [15, 16], and life cycle assessment [17]. The convolutional neural network was first applied in the field of images, and after achieving excellent performance in this field, researchers have successively proposed algorithmic models that apply it to video recognition, such as large-scale video classification [18], multimemory convolutional neural network [19], and tube convolutional neural network [20]. If this technology can be used to recognize technical basketball moves as they appear in the video, this would be an important application of deep learning in sport. Such practical applications hold great promise for basketball training. For example, it could be used to inform professional players, analysts, or basketball coaches about technical movements, and it could also assist referees in judging games on the court. Deep learning techniques such as wearing sensors on human joints to extract skeletal point location information for movement recognition have significant limitations, whereas it is easier and more efficient to recognize human movements in the dynamic video [21–23]. Therefore, a large number of researchers have investigated action recognition tasks through deep learning [24–26], by improving on the classical structures of convolutional neural networks to obtain frame-specific features and interimage timing information in videos.

Therefore, it is relevant to carry out work on the recognition of basketball technical movements through the use of deep learning methods. For all types of coaches and professional athletes, this technology will help them to better analyze the video in order to provide more effective coaching and training for their players.

## 2. Target Detection Method Based on Convolutional Neural Networks

**Figure 1:**
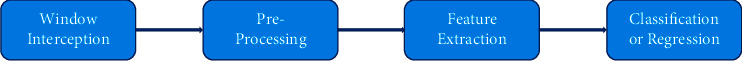
Traditional target detection algorithm.

The traditional target detection algorithm has roughly flowed as shown in Figure 1. The main process is to first intercept the image by means of a sliding window. The shape and size of the interception window vary due to the size of the target, which results in a large number of redundant windows [27]. Next, the information in the window is extracted for features. There are a number of methods for feature extraction, including methods based on shape and color according to rules specified for different tasks. This step of feature extraction is critical as algorithm performance is often causally related to a large extent to the quality of the extracted features. After feature extraction, the target within the window requires to be classified and determined. Commonly used classifiers include SVM and AdaBoost, and the final detection result is generated.

Liu et al. proposed a region convolutional neural network (R-CNN) algorithm based on deep learning, which used convolutional neural networks to recognize the images [28]. This research focuses on introducing R-CNN, fast R-CNN [29], and faster R-CNN [30].

### 2.1. R-CNN

**Figure 2:**
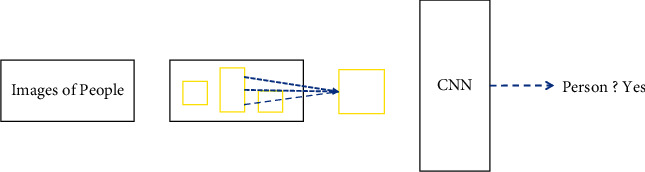
Framework of R-CNN.

Within the field of deep learning-related target detection, R-CNN is the first algorithm to apply convolutional neural networks to target detection. In contrast to traditional target detection algorithms, R-CNN first obtains a preselected box where the target may be located and performs feature extraction within the preselected box. This process is achieved through a selective search algorithm, and thus, window redundancy can be largely avoided, resulting in a definite improvement in the detection speed and accuracy of the network. As shown in Figure 2, the R-CNN acquires preselected frames from the input image and then transforms the image to make the preselected frames consistent in size for subsequent processing. Finally, it is fed into the network model, and the feature vector is output, which is used by the classifier to determine whether it belongs to a certain category.

Compared to sliding window target detection algorithms, R-CNN algorithms are faster, but there is still a large gap in real-time detection results. The main reason for this is that the feature extraction process involves a lot of redundant computations, as each candidate region of the image is convolved. In addition, image compression and image cropping operations are performed on each candidate region, and some of the image feature information is lost to a certain extent.

### 2.2. Fast R-CNN

Compared to the traditional loss function, fast R-CNN proposes a multitask loss function that combines the loss of classification and the loss of border regression and incorporates it into the CNN as a way to correct the position information of candidate frames.

The loss function includes classification loss and regression loss, which are shown in the following formula:

(1) $$\begin{array}{c} L\left(p,u,t^{u},v\right)=L_{c}\left(p,u\right)+\lambda \left[u\geq 1\right]\times L_{r}\left(t^{u},v\right), \end{array}$$

where *L*_*c*_ refers to classification loss.

(2) $$\begin{array}{c} L_{c}\left(p,u\right)=-\log\left(p_{u}\right), \end{array}$$

where *L*_*r*_ indicates regression loss.

(3) $$\begin{array}{c} L_{r}\left(t^{u},v\right)=\sum_{i\in \left\{x,y,w,h\right\}}{\text{smooth}}_{L_{1}}\left(t_{i}^{u}-v_{i}\right),\\ {\text{smooth}}_{L_{1}}\left(t_{i}^{u}-v_{i}\right)=\left\{\begin{array}{cc} 0.5\times {\left(t_{i}^{u}-v_{i}\right)}^{2}, & \left|\left(t_{i}^{u}-v_{i}\right)\right|<1,\\ \left|\left(t_{i}^{u}-v_{i}\right)\right|-0.5, & \text{other}. \end{array}\right. \end{array}$$

### 2.3. Faster R-CNN

The performance of fast R-CNN target detection algorithm is much improved compared to the R-CNN algorithm, but the bottleneck of fast R-CNN is the step of the selective search algorithm to generate the detection frame of the image. Faster R-CNN generates candidate frames by doing a region proposal network (RPN). At this point, the faster R-CNN is considered to be an end-to-end detection algorithm.

**Figure 3:**
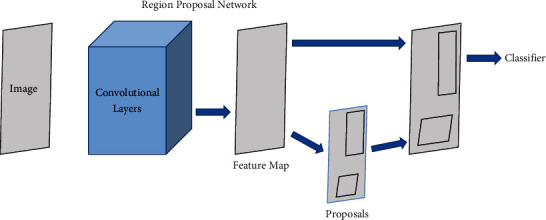
Framework of faster R-CNN.

As shown in Figure 3, faster R-CNN uses RPN to extract prediction frames on the feature map, map the prediction frames onto the feature map, and feed them to the end-to-end detection algorithm. The prediction frames are mapped onto the feature map and fed into the Fast R-CNN for classification and regression.

## 3. Video Action Recognition Based on 3D-CNN

In basketball training, when performing human action recognition, the input data are a sequence of video frames. Therefore, it is necessary to consider not only the representation of the action in space but also the sequence between the atomic actions of the action in the video frame sequence. The traditional 2D convolutional networks cannot handle the sequential order of atomic actions when dealing with action recognition in video. The ability to determine an action from just one frame in a real-world scenario is very limited. Features need to be extracted from a continuous video frame in order to classify actions more accurately. This problem can be solved in 3D convolutional networks.

### 3.1. Framework for Dual-Resolution 3D-CNN

**Figure 4:**
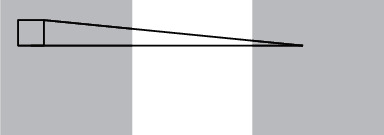
2D convolution operation.

**Figure 5:**
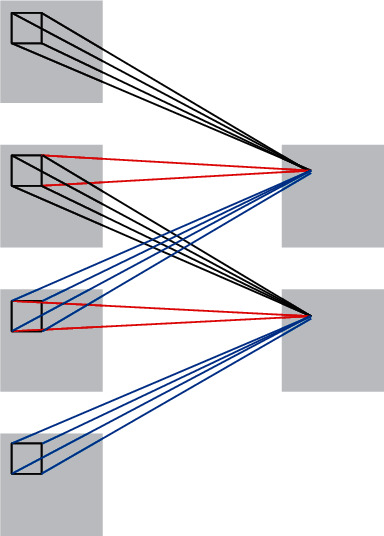
3D convolution operation.

The 3D-CNN (3D convolutional neural networks) has the ability to process continuous information between motion images by extending the traditional CNN to include a temporal dimension on 2D so that the video data contain both temporal and spatial dimensions. 2D convolutional operations are compared to 3D convolutional operations as shown in Figures 4 and 5.  Figure 5 captures the motion information in three consecutive frames by computing them in a temporal dimension of 3.

In the same way as 2D convolutional kernels, 3D convolutional kernels extract certain types of features from stacked video frames using a weight-sharing approach. To get a better feature representation, more types of features can be extracted by increasing the variety of convolutional kernels or adjusting the convolutional kernel weights compared to the same convolutional kernel in the whole video cube. This is because the deeper the network is, the more data are available in the feature map, and more types of abstract features need to be generated from the combination of shallow features.

The most straightforward way to speed up network training is to reduce the number of convolutional layers and fully connected layer parameters, but this reduces the performance of the network. In order to ensure the performance of the training without changing the size of the network, the high-resolution data can be downscaled to low-resolution data, and then, the training experiment can be conducted. The experimental results show that although the network is faster, the low-resolution input data still lose some feature information, and certain details of the high resolution have an impact on the recognition rate.

**Figure 6:**
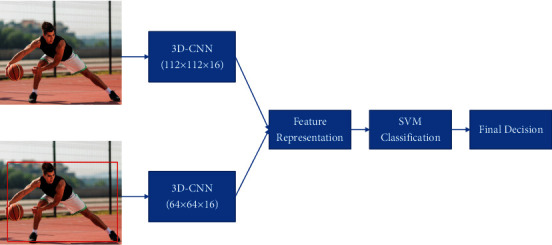
Framework of dual-resolution 3D-CNN.

As shown in Figure 6, the aim is to process successive technical action frames at two different resolution inputs and then fuse the features to further improve the recognition capability. A video clip with a resolution of 112 × 112 and 16 consecutive frames is used as input to the network. The upper stream is the original frame image after video preprocessing, and the lower stream is the cropped image after SSD target detection (circled in red). The lower stream is the cropped image after SSD target detection (circled in red), with a resolution size of 64 × 64.

A picture is composed of pixel points based on a mixture of the three primary colors of RGB in different proportions. When the original picture data are fed directly into the network, the amount of input data will be larger, increasing the training time. However, in the process of human action recognition, the color information utilization of the picture is relatively low, and grey scaling of the original image can be considered. This is because even in a greyscale image, each pixel of the image still has a certain range of variation and can still retain the vast majority of the information such as gradients in the original image. Using the network to process greyscale images can reduce the amount of computation to a certain extent, thus achieving more efficient use of memory. In basketball technical action recognition, differences in the background of the court and changes in lighting and the color of the person's clothing can have an impact on the recognition. By grey scaling the images, the effects of these distracting elements can be ignored to a certain extent, so grey scaling of technical action images is feasible.

### 3.2. Initialization Weighting Strategy

In order to improve the training efficiency, this research proposes a method to initialize the weights by combining temporal information with the spatial convolution of image frames. In the 3D convolution layer, the 3D convolution weight parameters are initialized by using ImageNet's 2D weight parameters.

#### 3.2.1. Mean Initialization

Since continuous image frames of technical actions have similar backgrounds, similar image information can be extracted through the following equation:

(4) $$\begin{array}{c} W_{t}^{3\;D}=\frac{W^{2\;D}}{T}. \end{array}$$

#### 3.2.2. Initialization by Scaling

According to equation (5), by introducing some diversity, any combination of constants can be used.

(5) $$\begin{array}{c} W_{t}^{3\;D}=a_{t}\times W^{2\;D}. \end{array}$$

### 3.3. Feature Fusion

A 3D-CNN network with different resolutions after the original frames and cropped frames, respectively, will result in a corresponding weight file. Using the weights as parameters for the model, the technical action frames are predicted separately to obtain a 1024-dimensional feature vector for the corresponding frame sequence. After extracting the features, the features are fused, which is done for subsequent classification and recognition of the fused features. There are many ways to fuse the features, but in this paper, we choose the form of equation (6). As long as the dimensions are the same, the dual-stream models can be fused at the corresponding positions, even if there are minor differences that can be solved by filling in the differences by means of translation. Therefore, this research will fuse the features of the dual resolution streams by summing the results of the two feature maps. Let the final feature representation for the input continuous technical action frame be *Y*. At the same spatial locations *i*, *j* and feature channel *d*, the equation for *Y* can be expressed as follows:

(6) $$\begin{array}{c} Y=X_{i,j,d}^{a}+X_{i,j,d}^{b}. \end{array}$$

**Figure 7:**
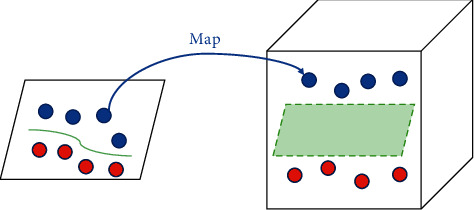
SVM maps features from linearly nonpartitionable to linearly partitionable.

As there are only six categories for classifying basketball technical movements and a total of 1800 video frame sequences, this sample size is suitable for application to a support vector machine (SVM). SVM is a statistical learning method based on the principles of structural risk minimization and VC dimensionality. The main logic of the SVM algorithm is to train with labeled training samples and output a hyperplane that is optimized and separates the majority of high-dimensional data while minimizing the overall risk of the structure. The distinction between two-dimensional point data is simple: there are an infinite number of straight lines between the two categories that allow the training data to be classified correctly. In general, straight lines that are too close to a sample of a class are not optimal for classification because the bounds of such classification are more sensitive to the noise signal of the data, resulting in poor generalization. For linearly divisible data, the SVM obtains the optimal bounds by minimizing the distance between the bounds and all the training samples. When the training sample data are linearly indistinguishable, the SVM converts the nonlinear low-dimensional data into linearly divisible in the higher dimensional space by mapping it to some extent by means of a kernel function. This operation then converts the feature vectors in the input space into separable vectors and finally obtains the best performing classification hypersurface in the higher dimensional space by linearly separable methods. This is illustrated in Figure 7.

### 3.4. Comparison of Experimental Results

The number of frames has an impact on the recognition performance when the scale of the input video frames is the same. If the number of frames is too small, for example, if only one frame is input, it degrades to an action recognition based on a single frame, which is based on one frame only without temporal information and does not perform well for continuous action recognition. If the number of frames is too high, it is necessary to consider whether the number of frames per video is sufficient and to consider using data augmentation to generate more training data when there are not enough data. Also, the higher the number of frames, the higher the training volume of the network model, and the longer the time required.  Table 1 shows the comparison of the recognition rate of the dual-stream 3D-CNN model for different frame counts of the dataset input in this study. From the experimental results, it can be seen that the algorithm achieves the highest recognition efficiency of 96.9% when 15 consecutive video image frames are input.

Taking 15 consecutive video image frames as an example, in order to verify the performance of dual-resolution 3D-CNN and single-resolution 3D-CNN for technical action recognition on basketball technical action video sets, this section compares the performance of the original frame 3D-CNN recognition algorithm and the cropped frame 3D-CNN recognition algorithm on basketball technical action video sets. From the experimental results, it can be concluded that the performance of the multiresolution 3D-CNN on basketball technical action recognition has been improved compared to the single-resolution approach of original frames and cropped frames, especially for jump shooting action and turning action with a recognition rate of over 95%. The results from the confusion matrix show that the experiments have some misclassification. For example, the turnaround and Sam Gord move, which may be due to some similarities in that both mislead the defender's defensive line before breaking through from the other side. There is also some confusion between the turn and the Dunbay move, probably due to the fact that there are few frames of combined ball movement between both technical moves, so there is some misclassification.

## 4. Conclusion

This study focuses on the action recognition problem in video basketball technology and proposes a recognition architecture that combines an SSD-based target detection algorithm and a multiresolution 3D-CNN for action recognition. In addition, the study focuses on the extensive use of convolutional neural networks in the field of deep learning. The main target detection algorithms and action recognition algorithms are also presented in detail. A series of data processing processes are used to create a dataset of 1800 basketball technical action videos based on practical technical actions that occur in the professional NBA, popular highlights, and self-published basketball instruction on short video platforms, and the evolution of actions under modern basketball rules. Finally, a dual-resolution 3D-CNN architecture-based video basketball action recognition algorithm was designed to build a CNN network for action recognition. The effectiveness of the algorithm was verified by comparing the original frames and cropped frames and SVM classification after fusing features on the basketball technical action dataset.

Although some progress has been made in this study on a home-made basketball technical action dataset, there is still a certain gap from practical application and many practical problems to be solved. In action recognition based on target detection, how to better identify technical actions if the character action involves certain occlusion and interference, or if multiple near-identical sized targets appear in the same shot. In video-based basketball technical action analysis, information can be used to include the attacker, the defender, and the interaction between the two. As there is positional information on the court such as the free-throw line and the three-point line, can this be used to indicate the position of the person and use this to infer the action. These have some promise in terms of finding new cues to achieve improved recognition. If these problems can be solved, then they can be used to improve the performance of technical basketball action recognition. Furthermore, the representation of high-level information and low-level features for technical action recognition draws on other issues previously investigated whether there are unique behavioral representations applicable to basketball actions and, in doing so, investigates better representations than the current features.

## Figures and Tables

**Table 1:** Comparison of experimental results.

Frame	Accuracy (%)
6	83.6
11	87.3
12	89.1
15	96.9

## Data Availability

The labeled dataset used to support the findings of this study is available from the corresponding author upon request.
